# Nutritional Status of Adolescents Attending the Iranian Secondary School in Kuala Lumpur, Malaysia

**DOI:** 10.5539/gjhs.v6n6p185

**Published:** 2014-07-29

**Authors:** Maryam Zarei, Huang MSL, Mohd Nasir Mohd Taib, Fatemeh Zarei

**Affiliations:** 1 Department of Nutrition and Dietetics, Faculty of Medicine and Health Sciences, Universiti Putra Malaysia, Serdang, Selangor, Malaysia

**Keywords:** adolescents, Body Mass Index (BMI), dietary intake, body image

## Abstract

**Introduction:**

The aim or this study was to determine factors associated with body weight status among Iranian adolescents in the two Secondary Schools run by the Embassy in Kuala Lumpur.

**Methods:**

A self administered questionnaire was used to assess socio demographic characteristics, physical activity, and body image. Dietary intake was recorded through individual interviews with the researcher. The Physical Activity Questionnaire for children (PAQ-C) was used to evaluate levels of physical activity of the adolescents.

**Results:**

One-third (32.2%) of respondents were of normal weight, 14.5% and 11.1% were overweight and obese respectively, while 18.6% and 23.6% were severe thinness and thinness respectively. While the distribution of obese respondents by gender was almost the same, overweight females (16.4%) exceeded overweight males (12.7%) and although more females were in the thinness category (24.7% compared to 22.7%), more males were severely thin (20.0%) compared to 17.1% of the females. Body weight status was significantly associated with age (p < 0.05), gender (p < 0.05) and grade (p < 0.05). Males had significantly higher physically activity scores than females (p < 0.05). Intake of all micronutrients were higher than Dietary Reference Intake (DRI), except for vitamins B1, B2, C, D and E, Folate, Zinc, Magnesium, Potassium, Calcium and Phosphorus. There was a tendency for the females to overestimate their weight and 72.6% of them expressed their desire to lose weight while 60% of the males wanted to gain weight. There was also significant association between body weight status and perception of ideal body size (p = 0.000) and healthy body size (p = 0.000).

**Conclusion:**

This study provides some information for the Iranian Secondary school to design intervention programs to improve the body weight status of their students.

## 1. Introduction

Adolescence is a very important period because during this time, many rapid physiological and physical changes, due in part to hormonal changes taking place and any hindrance to normal development can have severe implications on the future health of an individual. A detailed examination of dietary requirements by age shows that all nutrient needs during the teenage years are higher than at any other time in the lifecycle, and according to Story and Stan (2005) failure to consume a sufficient and balanced diet can suppress growth and postpone sexual maturation. The increasing prevalence of overweight and obesity in adolescence has become an alarming issue as it is found to be a key predictor for obesity in adulthood. It is now well documented that being overweight during adolescence is a risk factor in long-term health problems such as diabetes, cardiovascular disease and premature death (Haslam & James, 2005). Knowing that overweight and obesity are the major risk factors to development of non-communicable disease in later life, it is thus crucial to understand the potential factors associated with body weight status in adolescents.

In Iran, part of the increase in the prevalence of obesity is due to economic growth, and, like most fast developing countries, obesity and associated chronic diseases like heart problems are on the increase. The prevalence of overweight and obesity in adolescents aged 12-18 years were 10.9% and 11.3%, respectively, in one of the cities in Iran (Kelishadi et al., 2007). Meanwhile, in Malaysia the pattern is almost the same with Rampal et al. (2007) reporting the prevalence of overweight among secondary school students age 13-17 years to be 10.6% in males and 6% in females. The purpose of the present study was to determine the factors associated with body weight status among Iranian adolescents studying in Kuala Lumpur.

Malaysia, in the last few years has become a popular destination for Iranians seeking further education or overseas employment. The setting up of a special Iranian school to cater for the needs of the Iranian population in Kuala Lumpur (KL) is testimony to this. Many families come to Malaysia with their families. According to the Iranian embassy there are now more than 50,000 Iranians living around KL. The children of these families study at the Iranian school and their eating habits are basically formed away from their home country. There is, therefore, a need to know their habits in order to know if the food habits formed away from their home country affect their nutritional status.

## 2. Material and Methods

### 2.1. Study Design and Population

This was a cross-sectional research design aimed at assessing factors associated with body weight status among Iranian adolescents studying in the lower Secondary and High School in Ampang Point, Kuala Lumpur. A total of 296 Iranian female and male adolescents aged 12.0 to 17.9 years in the Iranian school were chosen as respondents in the present study. The sample size was determined using this formula: n=Z^2^_1-α/2_ P (1- P)/d^2^ = 1.96 2 (25%) (1-25%)/0.052 which is equivalent to 288.

### 2.2. Measurement Tools

Anthropometric measurements, body weight and height were taken and Body Mass Index (BMI) was calculated to determine the weight status of the respondents. The World Health Organization (2007) cut off was used for calculating the Body Mass Index for age in 15-19 years. Each respondent was measured twice for both height and weight and average of both measurements was used. The questionnaire was self-administered with three major sections including socio demographic information, physical activity record and body image. Dietary intake was recorded through individual interviews with the researcher. In the socio demographic section, adolescents were asked to answer 11 questions including their age, sex, grade level, parents’ education, parents’ occupation, number of siblings, length of residence in Kuala Lumpur, living arrangement (alone/with family/with a single parent), household income, pocket money and academic performance. Academic performance was cross referenced with the academic record office in the schools. The Physical Activity Questionnaire for children (PAQ-C), which was adopted from Kowalski et al. (2004), was used to determine the level of physical activity of the adolescents. The questionnaire has 10 items. Item 1 was on leisure time activity of the adolescents during the last 7 days where the respondents were required to respond to an activity checklist and was scored on a 5-point scale ranging from “NO” activity being scored as 1 and “7 times or more” being scored as 5. Item 2 to 8 were on activities of the respondents during physical education (PE) class, recess, lunch, right after school, evenings, weekends, and leisure times. For item 9, the respondents were asked about the frequency of participating in daily physical activity (for example: playing sports, games and dancing) in the previous week. A score of 1 was assigned to “none” and a score of 5 was assigned to “very often”. Item 10 was asked to identify respondents with common activities during the previous week. In the present study the internal consistency of this assessment calculated from an independent sample of 24 adolescents was Cronbach’s alpha= 0.75. The questionnaire on the body image consisted of two sections which included perception of body size and perception of body weight. A total of three questions were used to assess the perception of the adolescent’s current body weight status, current body weight satisfaction and the desire to change body weight (either to gain or lose weight). These questions were adopted and modified from Simko (1989). For assessing the Perception of Body Size we used three questions. The respondents were shown a series figures from the “Contour Drawing Rating Scale”, which was adapted from Thomson and Grey (1994). In the “Contour Drawing Rating Scale”, the 9 sub-figures were each given a number representing different degrees of weight status.

Dietary intake was based on the 24-hour dietary recall for 2 days comprising one on a weekday and the other in the weekend, a food frequency questionnaire (FFQ) and a meal pattern questionnaire. The 24-hour dietary recall method was used to estimate the mean nutrient intake. All the foods and beverages, including cooking methods, brand names of the processed foods and quantities of the food consumed were obtained through interviews. Respondents had to estimate the quantity of food consumed in standard household measures using standardized cups and spoons. For composite dishes, the amount of each ingredient used in the recipe was estimated by researcher. All food records were coded by software. Household measurements recorded were converted into food weights in grams based on a list of food weights according to Food Composition Table by (Tee et al., 1997) and entered into Nutritionist ProTM for analysis. Obtained data were analyzed based on the Malaysian food database using Nutritionist ProTM software (Axxya Systems, 2000). If cooked dishes like certain Iranian foods were not found in the database of Nutritionist Pro™ Software, the investigator made an original recipe from standard cook books for each dish (per 100 gram) and then the quantitative information was entered to Nutritionist Pro software. The software was then used to calculate the average energy and nutrient intakes of the respondents. The data obtained were entered to SPSS to be analyzed. Then the average of energy and nutrients for the two days 24-hour dietary recall were compared to Dietary Reference Intake (DRI). Energy and some nutrient intakes such as (protein, fat, fiber, vitamin A, C, D, thiamine, riboflavin, niacin, pyridoxine, folic acid, calcium, magnesium, iron, sodium, zinc, manganese, potassium, phosphorus) were assessed based on Dietary Reference Intake (DRIs). The Dietary Reference Intake (DRI) was used to assess the nutrient intake of this population (age 9-13 males and females and also 14-17 year old males and females).

### 2.3 Pre-Testing

Pre-testing for the questionnaires was carried out on May 08, 2009, among 24 adolescents (12 male and 12 female) from the Iranian Secondary School in Kuala Lumpur. The pre-test was conducted in order to ensure that items and questions in the questionnaire can be understood in term of its content. The pre-test revealed that the respondents could understand most of the questions. Only a few questions that dealt mainly with physical activity and body images needed further explanation. Consequently, some modifications were done in the questionnaire. The reliability of pre-testing questionnaires were measured by Cronbach’s alpha = 0.89.

### 2.4 Ethical and Legal Approval

This study was approved by the Medical Research Ethics Committee, Faculty of Medicine and Health Sciences, Universiti Putra Malaysia. Parents’ informed consent was also obtained before the commencement of the study.

### 2.5 Data Collection

Data for the study were collected by using questionnaire which was written in Persian. The researcher assessed anthropometric measurements of weight and height in the school and collected the self-administered questionnaires by the respondents on the same day. All anthropometric measurements (height and weight) were assessed by the researcher. Respondents were interviewed by the researcher on two separate days to record their dietary intake. Data collection was conducted in May and June of 2009.

### 2.6 Statistical Analysis

All the results were analyzed using the Statistical Package for the Social Sciences (SPSS version 17.0 software). Descriptive statistics such as frequencies, means, standard deviations and percentages were used to describe variables. The T-test was used to determine any significant difference in dietary intake, body image, physical activity and BMI between male and female respondents. For all categorical variables Chi-square was used in this study. Pearson Product-Moment Correlation was used to determine association of two continuous variables. Multiple linear regression analysis was performed to measure the amount of influence a predictor variable had on a dependent variable. Results were considered to be significant when the observed significance level was p < 0.05.

## 3. Results

### 3.1 Socio-Demographic Characteristics of Respondents

A total of 296 adolescents (150 males and 146 females) with a mean age of 14.33 ± 1.80 years participated in this study. Table 1 shows the demographic background of the respondents. About one third (33%) of them were aged 12 to 13 and the other 77% were thirteen years and older. As is evident from Table 1 parents of the respondents were highly educated with fathers being better educated than mothers. Almost half of the mothers were housewives, while another 48% were students pursuing their post graduate studies in Malaysia. Most fathers (60.1%) were post graduate students while 39.9% were working in Kuala Lumpur. Basically the respondents came from small families with an average of only 1.8 siblings and in fact a quarter of them had not sibling at all. Almost all of them lived with their families and on the average had been in Malaysia around 20 months. Average monthly household income was RM 2,498.85 and pocket money received ranged from RM 10 to 50 per week. On a scale of 0 to 20, academic performance of the respondents average 15.5 points with more than half scoring 16 to 20 and 47.6% scoring 11 to 15 points.

**Table 1:** Socio-demographic characteristics (n = 296)

Characteristics	Respondents	
	n (%)	Mean±S.D
Education Level		
Secondary School	150 (50.7)	
High School	146 (49.3)	
Gender		
Male	150 (50.7)	
Female	146 (49.3)	
Age Groups (Years)		14.33±1.8
12.0-13.0	100 (33)	13.1±1.49
>13	196 (77)	16.36±1.4
Mothers’ Educational Level		
High school	19 (6.5)	
Diploma	43 (14.5)	
Bachelor	83 (28)	
Master	113 (38.2)	
PHD	38 (12.8)	
Fathers’ Educational Level		
High school	18 (6.2)	
Diploma	6 (2)	
Bachelor	38 (12.8)	
Master	75 (25.3)	
PHD	159 (53.7)	
Mothers’ Occupation		
Housewife	137 (46.3)	
Working	17 (5.7)	
Student	142 (48.0)	
Fathers’ Occupation		
Student	178 (60.1)	
Working	118 (39.9)	
Number of siblings		1.81±0.53
0	72 (24.3)	
1	209 (70.3)	
≥2	15 (5.40)	
Living arrangment		
Alone	8 (2.7)	
With Family	288 (97.3)	
Duration of Time in Malaysia (Months)		19.95± 10.18
<12	96 (32.5)	
13-24	120 (40.5)	
25-36	64 (21.6)	
>37	16 (5.4)	
Pocket money per week(RM)		25.77±10
10-20	132 (44.6)	
21-30	30 (10.2)	
31-40	96 (32.4)	
41-50	38 (12.8)	
Total Monthly Household Income (RM)		2498.85±474.88
1001-1500	15 (5.1)	
1501-2000	31 (10.4)	
2001-2500	107 (36.1)	
2501-3000	57 (19.3)	
>3001	86 (29.1)	
Academic performance		15.53±2.3
0-5	0 (0)	
6-10	5 (1.70)	
11-15	141 (47.6)	
16-20	150 (50.7)	

### 3.2 Anthropometry

As is displayed in Table 2, mean weight, height and BMI-for-age of the respondents were 45.21 ± 10.42 kg, 153.25 ± 9.11 cm and 19.12 ± 3.43 kg/m^2^ respectively. The mean BMI for females (19.49 ± 3.65 kg/m^2^) was higher than that of males (18.76 ± 3.17 kg/m^2^). Respondents’ BMI were compared to WHO BMI-for-age reference (WHO, 2007) and divided into five categories. One-third (32.2%) of respondents were normal weight, 14.5% and 11.1% were overweight and obese respectively, while 18.6% and 23.6% were severe thinness and thinness respectively. While the distribution of obese respondents by gender was almost the same, overweight females (16.4%) exceeded overweight males (12.7%) and although more females were in the thinness category (24.7% compared to 22.7% in males), more males were severely thin (20.0%) compare to 17.1% of the females.

**Table 2:** Distribution of respondents by consumption of breakfast (n = 296)

Consumption of breakfast	Male (n=150)	Female (n=146)	Total (n=296)
	Frequency of Breakfast consumption (n=296)		
Had Breakfast	141 (94)	81 (55.5)	222 (75)
Did not have breakfast	9 (6.0)	65 (44.5)	74 (25)

	Number of Time for Breakfast (n = 222)		
	Male (n = 141)	Female (n = 91)	Total (n = 222)
Fixed	75 (57.3)	58 (63.7)	133 (59.9)
Different	56 (42.7)	33 (36.3)	89 (40.1)

	Frequency of intake (n=222)		
Alone	67 (47.5)	47 (47)	114 (46.5)
With family	72 (51.1)	41 (51)	113 (51)
Friends	2 (1.4)	12 (8.4)	17 (4.6)

### 3.3 Physical Activity

The findings showed that males and females are involved in different activities. While 10% of the males swam and played football every day during the previous week, none danced, cycled, did aerobics, played volleyball, basketball or badminton, walked for exercise, jogged or played tagging. In the contrast, 17.8% of the females danced more than 7 times during the previous week. In addition, the majority of the females did not or seldom participated in activities such as swimming, football, tagging, basketball, skipping, and volleyball, aerobics, cycling or running. Part of the reason was the lack of facilities available to them. In general, most of the respondents did not participate in any physical activity during the previous week and only a few males and females were active in swimming and skipped 5-6 times the previous week. However, 14.4% of the females were active in dancing which can be done in the confines of their home or room (3–4 times the previous week) and 7.6% of the males swam 3–4 times during the previous week. This is only possible for those who lived in condominiums with swimming pools. On weekends, there was no report of any activity in excess of twice for both the male and female respondents. Around 85.3% of the males and 78.1% of the female respondents reported not being involved in physical activity during the previous weekend. Only 14.7% of the male and 21.9% of female respondents participated in some form of physical activity once during the previous weekend. Distribution of the respondents by physical activity levels reflects the lower involvement of females. As is evident in Table 3, while only 76.7% of the males recorded low physical activity, 84.9% of the females were in the same category and 6.6% and 2.7% of the males and females respectively reported high physical activity. The mean physical activity level of the male respondents was 1.3 ± 0.58 which was significantly higher (t = 2.0, p<0.05) than for that of the female respondents (1.17 ± 0.45).

**Table 3:** Food frequency score

Food	Score	Food	Score
A. Highly consumed foods (Score: 80.0 - 100.0)			
Cooking oil	92.82	Tea	81.69
Cooked Rice	89.21	Meat	81.00
White bread	88.11	Sandwich	80.00
Chips	87.41		
B. Moderately consumed foods (Score: 60.0 - 79.9)			
Cookie	65.77	Tomato	61.55
Cake	65.55	Lettuce	61.51
Egg	64.30	Apple	61.50
Cooked potato	63.42	Sugar	61.01
Ice cream	62.45	Fast food	60.14
Cookie	65.77	Coffee	60.10
Cake	65.55	Tomato	61.55
C. Less consumed foods (Score: 0 - 59.9)			
Cooked corn	57.34	Carrot	38.18
Chocolate	55.51	Lentil	38.14
Chocolate milk	55.51	Mango	37.57
Nut	55.04	Green Pepper	37.34
Honey	53.61	Lemon	36.42
Jam	52.61	Tangerine	36.26
Orange	52.60	Mangos teen	36.23
Orange juice	52.43	Yogurt	34.42
Chicken	52.00	Full Cream Milk	33.28
Tomato sauce	51.87	Gourd	32.62
Pizza	51.18	Watermelon	32.02
Cheese	48.61	Low fat Milk	31.44
Iranian bread	47.50	Plum	30.94
Fish	46.31	Chick pea	30.35
Red bean	45.07	Pear	28.88
Banana	43.51	Butter	26.97
Pasta	40.78	Dried fruits	24.07
Rambo tan	40.42	Grape	23.20
Egg plant	40.37	Pine apple	22.31
Spinach	39.87	Soya	18.40
Whole grain bread	39.22	Beet	15.64
Green bean	38.27		

### 3.4 Dietary Intake and Food Frequency

Dietary intake was based on the 24 hour dietary recalls and in this study all the 11 to 13 year olds did not meet their energy and vitamin D requirements. Meanwhile only 79%, 59%, 66%, 52% and 89% met their dietary reference intakes (DRI) for vitamins C, E, B1, B2 and dietary fibre respectively. On the other hand in the 14 to 17 age group 75% of respondents met their DRI for energy, 92.3% for vitamin C, 48.6% for vitamin E, 59.2% for vitamin B1, 64.8% for vitamin B2, 60.7% for folate, 51% for vitamin B12 and 94.9% for dietary fibre (94.9%). However all 14–17 year olds met their DRI for vitamin D. Meanwhile, in both groups more than half of the respondents’ intake of vitamin A was sufficient. With the respondents aged 11 to 13, 75% and 72% of them did not meet the DRI for protein and carbohydrates respectively. In the case of the 14 to 17 age group, 87.8% and 65.3% did not meet their protein and carbohydrate DRI, respectively. In general, all of the respondents in age group 11–13 and 75% of those in the 14–17 age groups respectively did not have adequate energy intakes.

There was a significant association between daily intake of protein, vitamin A, C, B1, B2, niacin, calcium and iron with body weight status. While, there was no association with energy intake, carbohydrate, fat, fibre, vitamin D and calcium (Table 4). The reason could be that consumption of fruits and vegetables, milk and dairy were lower than DRIs and Khor (2003) revealed that micronutrient deficiency is also common among Asian children

**Table 4:** Correlation between body weight status and dietary intake

Variable	r	p-value
Protein (g)	0.10	0.04*
Vitamin A (µg RE)	0.16	0.001**
Vitamin C (mg)	0.22	0.000***
Vitamin B1(mg)	0.11	0.000***
Vitamin B2 (mg)	0.19	0.000***
Niacin (mg NE)	0.11	0.047*
Calcium (mg)	0.17	0.02**
Iron (mg)	0.15	0.04**

* Significant (p<0.05),

** Significant (p<0.01),

*** Significant (P<0.001).

In this study most of the respondents (45.9%) chose Iranian foods like (Chicken kebab, rice with lamb, rice with chicken, rice with lamb and vegetables and fish) while 43.7% were more likely to choose fast foods including from KFC, MacDonalds, Burger King and Pizza Hut. Only 2.7% chose Malaysian foods such as noodles, *Nasi goreng* (fried rice), *Roti* (local bread) and *Nasi ayam* (chicken rice) and only 7.7% of respondents claimed that they did not have a preference in their choice of foods. The percentage of respondents who chose fast food and Iranian foods were much higher than others. Therefore it was not surprising that the food frequency score actually showed that consumption of rice, white bread, oil and chips was high while cucumber, potatoes, green leafy vegetables, cookies, cakes, eggs, cooked potatoes, ice cream, tomato, lettuce, apple, sugar, fast food, coffee were in the moderately consumed foods and chicken, fish, some dairy foods such as cheese, yogurt and milk, fruits and some vegetables, pizza and pasta were among the less frequently consumed food. Since the preference of the respondents is for Iranian and fast foods it could affect their dietary intake. Iranian foods which are favored are indeed difficult to find in Kuala Lumpur and the only way is for their families to prepare it. Many of the parents are busy graduate students who may find juggling their time between demands of graduate school and preparing meals for the family difficult. Therefore fast foods would be the other choice. These respondents have not adapted themselves to eating readily available more nutritious local foods.

### 3.5 Body Image

**Figure 1:**
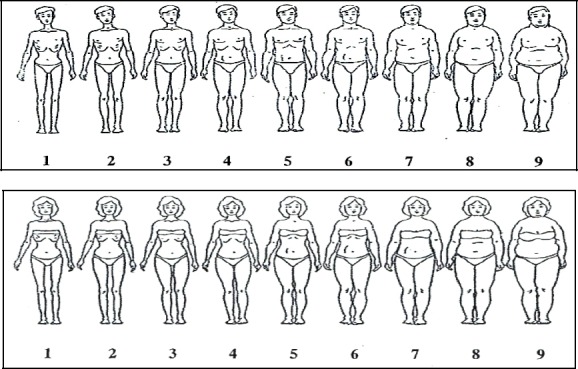
Series figure of ‘Contour Drawing Rating Scale” (Thompson & Gray, 1994)

More than one-third of the respondents (43.6 %) perceived themselves as having normal weight while 14.9% perceived themselves as being underweight. At the same time 41.5% of the respondents perceived themselves as overweight and obese. In the present study there was no association between perception of body weight status and current body weight status. More males perceived themselves as being underweight than females, whereas more females perceived themselves to be overweight/obese. According to Figure 1, 38% of the males chose the 4^th^ figure as their current body size, 60.7% chose the 5^th^ figure as their ideal body size and 55.3% chose the 6^th^ figure as the healthy body size. None of the male respondents chose Figures 8 and 9 as current, ideal or healthy body sizes. Meanwhile, 8% of the male respondents chose Figure 7 as their ideal and another 8% as their healthy body size. At the same time, 28.7% of male respondents chose Figure 4, 38% of them chose Figure 5 and only 11.9% of them chose Figure 3 as their current body size. The mean values for perception of current, ideal and healthy body were 4.78 ± 1.08, 5.47 ± 0.64, and 6.32 ± 0.76, respectively.

In the present study there is no relationship between current body size and body mass index. However, there was an association between ideal and healthy body size with body weight status among adolescents. The majority of both male and female adolescents were concerned with their body size.

### 3.6 Factors Associated with Body Weight Status

A total of 16.4% of females were overweight compared to 12.7% in males. In contrast, 20% of males were severely thin compared to 17.1% of the females. Chi-square test found significant association between BMI and gender (χ2 = 11.188, p < 0.01). Significantly more females were overweight/obese than males (p < 0.05). Chi-square test also reported significant association between body weight status and grade (level in school) (χ2 = 34.172, p < 0.05). While Pearson correlation test showed significant relationship (r = 0.23, p < 0.05) between age and weight status, duration of stay in Malaysia, number of siblings, parent’s education level, monthly household income, academic performance, parent’s occupation, weekly pocket money and living arrangement were not significantly associated with weight status. Pearson correlation found no significant association between body weight status and physical activity level. Table 4 shows correlation between body weight status and dietary intake and in this study BMI of respondents increased significantly with increased daily dietary intakes of protein, vitamins A, C, B_1_, B_2_, and niacin. There was no significant association between the perception of body weight status and current body weight status (χ^2^ = 9.22, p > 0.05). Overall, there was significant but weak association between body weight status and perception of ideal body size (r = 0.269 and p = 0.000) and healthy body size (r = 0.250, p = 0.000).

### 3.7 Multivariate Linear Regression

Multiple Regression by stepwise method was employed to identify significant predictors associated weight status. All independent variables including socio demographic indicators, physical activity, body image, dietary intakes were entered in the multivariate analysis. For categorical variables such as sex, mother’s job, father’s job, binary (dummy) variables were used before entering into analysis. Table 5 shows the R-squared (Coefficient of Determination) of 0.083 represented that the two predictor variables accounted for 8.3% of the variation in body weight status. Furthermore, result of this analysis showed that R (Multiple Correlation Coefficient) obtained was 0.29. Based on the Guildford’s rule of thumb, the R value obtained showed that there was a moderate relationship between the two predictor variables (age and physical activity score) to body weight status.

**Table 5:** Association between perception of body weight and current body weight status (n = 296)

Perception of body weight status	BMI of age				

	Severe thinness n (%)	Thinness n (%)	Normal n (%)	Overweight n (%)	Obese n (%)
Under weight	10 (22.7)	12 (27.3)	13 (29.6)	6 (13.6)	3 (6.80)
Normal weight	20 (15.5)	31 (24.0)	42 (32.6)	19 (14.7)	17 (13.2)
Over weight/Obese	25 (21.4)	27 (20.5)	40 (33.3)	18 (13.7)	13 (11.1)

In addition, F-statistics (10.84) was significant and the corresponding p-value is highly significant (p < 0.05). It can be concluded that the regression model fit the data at 0.001 level of significance. Furthermore, Table 5 shows that age (t = 3.98, p = 0.000) and physical activity score (t = -2.01, p = 0.042) was significantly associated with body weight status at p < 0.05. The largest beta coefficient is 0.22 which is for age. This showed that age made the strongest contribution to explain the body weight status among adolescents, when the variance explained by other predictor variable in the model was controlled. The beta value for physical activity score was “-0.11”. In conclusion, only two variables were found to be significant in explaining body weight status indicators. The equation of the multiple regression model derived from analysis was as follows: BMI = 14.65+0.44*age+(-0.55)*physical activity score.

## 4. Discussion

Only one-third of the Iranian adolescents studying in the Iranian School in Kuala Lumpur were of normal weight with more than a quarter being overweight or obese confirming the worldwide concern for the increasing obesity problem both in children as well as in young people. It would also be important to note that because they had only been in Kuala Lumpur for an average of less than two years the weight status was not necessary a reflection of their life in a new country but rather would have had their foundation in their home country of Iran. Therefore comparing their nutritional status with Iranian studies may be more appropriate. Montazerifar et al. (2009) reported that the prevalence of underweight, overweight and obesity of adolescents aged 14-18 respectively in Sistan and Baluchistan (South East of Iran) were 16.2%, 8.6% and 1.5% respectively. In the present study 11.1% were obese and 14.7% overweight due in part to the fact that these adolescents were from higher income and better educated families. The findings of the present study also showed that severe thinness was higher in males than females (p < 0.05), while, the prevalence of overweight was higher in females than males (p < 0.05, Table 2), which is consistent with studies by Rashidi et al. (2007) in Teheran and Aounallah-Skhiri et al. (2008) who reported that the prevalence of underweight was higher in males and overweight was higher in females. In contrast, in Malaysia Rampal et al. (2007) reported that the prevalence of overweight was significantly higher in the male (10.6%) compared to female (6.0%) in-school adolescents. Physical activity helps to expand and maintain healthy bones, muscles, and joints in early life; to enhance flexibility, balance, and survival for all ages; and to prevent or impede the development of high blood pressure, cardiovascular disease, and diabetes in adults (CDC, 1997). Physical activity also increases the risk of becoming overweight, which is more and more recognized as a serious public health issue affecting children and adolescents (Troiano, 2002). In this study physical activity among these adolescents was dismal due in part to the weather which is hot and humid all year round and the lack of facilities for physical activities. Culturally even if facilities were available Iranians are not used to exercising in places where they is no segregation by gender. Furthermore most of the adolescents are indeed children of graduate students who may be on limited budgets and joining clubs where children can have access to facilities may be limited. The school also does not make involvement in physical education compulsory and many of the adolescents actually reported absenting themselves from physical education classes in the curriculum. The types of activities the respondents were involved in differed greatly by gender with more males involved in outdoor activities. It is also not surprising that males report being significantly more active physically than females because being in a new country control of movement of adolescent females would be stricter. Low physical activity, as reported in the present study, means higher sedentary time and according to Elgar et al. (2005) and Anderson et al. (1998) weight status and physical activity may not be inversely related during adolescence. It is the amount of inactive or sedentary time, rather than hours of physical activity, that is a more accurate indicator of weight status in adolescents. Unfortunately the present study did not investigate sedentary time. Nonetheless it is often pointed out that those adolescents who have low physical activity score, have high body weight status (Barkery et al., 2000). This partly explains the higher prevalence of overweight and obesity among adolescents in the present study. In addition, age also plays an important role in body weight status. Jackson et al. (2007) found that when age increase, BMI also increases in adolescents. This is confirmed by Kosti and Panagiotakas (2006) who reported that the values of BMI show a trend to increase in all ages in both gender.

Significant difference between energy, protein, carbohydrate, vitamin B1, B2 and niacin among females and males consistent with a Malaysian study by Foo et al. (2006) who found that among Malaysian adolescents, the intake of energy and most nutrients were below that of the Malaysian recommended nutrient intake levels, with the exception of vitamin C and niacin. The boys had higher intakes for vitamin A, thiamine, niacin and vitamin C than the girls in their study. Similarly, Mirmiran et al. 2003 revealed that there were major gender differences in the mean daily intakes of energy, protein, carbohydrate, fat, fiber, cholesterol, iron, calcium and phosphorus. In addition, Bidad et al. (2008) and Oner et al. (2005) also reported mean intakes of niacin, pyridoxine, folic acid, pantothenic acid, vitamin E, calcium, magnesium, phosphorus, potassium, sodium and zinc being lower than the estimated average requirement (EAR) in male and female Turkish adolescents. Furthermore the respondents have only been in Malaysia for a short time, perhaps not long enough to adapt and accept Malaysian foods which are easily available and at a lower price than the food they preferred to eat.

In this study the majority of both male and female adolescents were concerned with their body size and understandingly so because adolescence is a period when they begin to be more conscious of themselves. More boys than girls thought of themselves as underweight, whereas more girls considered themselves to be overweight, consistent with previous findings on adolescents’ self-perception of body weight in China (Wang et al., 2009; Jin et al., 2008). In the present study there is no relationship between current body size and body mass index. However, ideal and healthy body size was associated with body weight status confirming the fact adolescents are more conscious their body size and societal norms. The finding that self-image perception is associated with weight status only in girls implies that they may be more aware of their body size than boys at this age, although overweight and obese boys and girls appear to suffer similar levels of body dissatisfaction. This finding is consistent with many other studies (Pruzinsky & Cash, 2004; Newman et al., 2006; Duncan et al., 2006). Studies have indicated that the maturation process brings about body image differences in adolescents (Mirza et al., 2005; Crow et al., 2006), with the female adolescents showing such concerns at an earlier age compared to male adolescents because they reach puberty earlier. Male adolescents selected larger figures as their current, ideal and healthy body size compared to female adolescents across all BMI status. While males selected a larger figure than their current body size as ideal body sizes, female adolescents selected a smaller figure than their current body size across all BMI status. This shows that females prefer thin body sizes and males prefer larger body sizes. This finding is consistent with many other studies (Wang et al., 2009; O’Dea et al., 2001, Khor et al., 2009). This could be due to cultural norms, media influences, religious and dietary practices among both sexes.

The present study found significant associations between the BMI and sex, age, dietary intake, and body image. However, multiple regression analysis showed that the physical activity was the factor most significant for predicting the body weight status of these sample Iranian students in school. This indicated that increasing their physical activities will reduce their respective BMI values. The findings of this study can provide baseline data for designing nutritional programs for the prevention and control of obesity among the Iranian adolescents. The results of this study should be shared with the school authorities including the teachers so that intervention programs can be planned and executed. It should also be noted that when addressing the needs of young Iranians in Malaysia special attention must also be paid to gender differences as well as cultural values especially those which are not practiced in Malaysia (for example the segregation of males and females in exercise facilities). Further research covering adolescents of various socioeconomic levels should be conducted in order to get a better picture of the situation among Iranian adolescents.

## 5. Conclusion

Adolescents are future leaders of a nation and healthy adolescents are fundamental to nation’s development. Adolescence is also a period when they form lasting eating habits that can affect their health in the future. Being in school provides the school with an opportunity to contribute to the adoption of healthy lifestyles. It is therefore important for school-based intervention programs that can be conducted to improve the nutritional status of their children. For a start it should be mad compulsory for all students to participate in physical education classes. Furthermore programs on improved dietary habits could be incorporated into sciences with emphasis on the selection of easily available local foods. Intervention programs to promote healthy body image can be developed among young girls to reduce their vulnerability towards distorted body image in later years. This study is only limited to the Iranian adolescents attending the Iranian School in Kuala Lumpur and should not be taken to represent all Iranian adolescents.
